# Computed tomography dosimetry with high‐resolution detectors commonly used in radiotherapy — an energy dependence study

**DOI:** 10.1120/jacmp.v16i5.5302

**Published:** 2015-09-08

**Authors:** Mario Liebmann, Bjoern Poppe, Heiner von Boetticher

**Affiliations:** ^1^ University Hospital for Medical Radiation Physics Carl von Ossietzky University, Campus Pius‐Hospital Oldenburg Germany; ^2^ Center for Radiology and Nuclear Medicine – Academy of Radiation Protection Klinikum Links der Weser Bremen Germany

**Keywords:** dosimetry, ionization chambers, computed tomography, CBCT, energy dependence

## Abstract

New methods of dosimetry in computed tomography (CT) X‐ray fields require the use of high‐resolution detectors instead of pencil‐type ionization chambers typically used for CT dose index (CTDI) measurements. This paper presents a study on the suitability of a wide range of ionization chambers, diodes, and a two‐dimensional detector array, used primarily in radiation therapy, for CT and cone‐beam CT dosimetry. Specifically, the energy dependence of these detectors from 50 kVp up to 125 kVp is reported. All measurements were performed in reference to a calibrated diode for use in this energy region. The radiation quality correction factors provided by the manufacturer were used, depending on the measured half‐value layer (HVL) for the particular X‐ray beam. Our study demonstrated the general usability of thimble ionization chambers. These thimble ionization chambers showed a maximum variation in energy response of 5%. Ionization chambers with even smaller sensitive volume, and which exhibit similar variation in energy dependence, can be used if higher spatial resolution is required. Furthermore, the investigated detectors are better suited for dosimetry at CT and CBCT units than conventional large volume or flat detectors, due to their rotational symmetry. Nevertheless, a flat detector can be used for certain measurement tasks, such as the acquisition of percent depth‐dose curves or beam profiles for nonrotating beams, which are important for beam characterization.

PACS numbers: 87.57.uq, 87.56.Da, 87.57.Q‐

## I. INTRODUCTION

Dosimetry for CT devices is mostly limited to measurements of so‐called computed tomography dose index (CTDI) values and various dose conversion factors.^1^, ^2^, ^3^, ^4^ These conversion factors are usually obtained with Monte Carlo simulations, which are calibrated themselves again with CTDI measurements.

According to the international (IEC 60601, EUR 16262) and national codes of practice (FDA amendments, DIN 6809‐3), the definition of the ${\text{CTDI}}_{100}$ is expressed as follows:

(1) $$CTDI_{100}=\frac{1}{nT}\int_{-50mm}^{50mm}D(z)dz$$

where *nT* is the total nominal collimation and D(z) the axial dose profile. The quantity ${\text{CTDI}}_{100}$ serves as the primary dose descriptor for computed tomography.^5^, ^6^, ^7^, ^8^, ^9^ Its value is measured within a PMMA phantom of 16 cm (head phantom) or 32 cm (body phantom) diameter and 15 cm length with an ionization chamber of 10 cm length.

For new 256 slice CT scanners, the X‐ray cone beam already exceeds this integration length without even consideration of scattering.^10^, ^11^ Furthermore, the human torso exceeds the body phantom length by a factor of more than two. Therefore, absorbed dose components from scattering inside the patient are not correctly accounted for in this concept.^(12)^

Additionally to the enlargement of the scanning beam, the routinely used scanning protocols are becoming more complex. Now common, tube current modulation (TCM) is utilized to ensure homogeneous image quality for scans of body regions with large variations in diameter, or to spare sensitive organs by reducing the tube current for certain arc segments within the tube rotation. These new technologies complicate the interpretation of the associated CTDI.^(13)^

Further dosimetry challenges result from the diversity in scanning techniques such as longitudinal and angular TCM, as well as variable pitch and z‐collimation like the new “rolling collimator”. The transfer of the CTDI concept also is problematic for scans without couch increment like perfusion studies or cone beam CTs in general. Therefore, new nonstandard measurement protocols for CTDI and CT dose profiles with small sensitive volume ionization chambers instead of pencil‐type ionization chambers in longer dosimetry phantoms were introduced.^14^, ^15^, ^16^, ^17^, ^18^

General X‐ray radiography ionization chambers, intended to measure incident doses, are usually plane parallel flat chambers with a relative large volume. In contrast, for dosimetry in CT fields, axially symmetric probes are needed and, especially for dose profile measurements, a small volume is required. Additional parameters needed for modeling X‐ray beams in Monte Carlo calculations, such as geometric efficiency, can only be determined using small volume detectors.^(5)^

In previous studies, several ionization chambers designed primarily for dosimetry of megavoltage photon beams have been investigated in terms of their usability in X‐ray therapy.^19^, ^20^, ^21^ These studies showed the general feasibility of these detectors for kV dosimetry, provided that detector‐specific correction factors for the spectral composition of the radiation fields are taken into consideration. Snow et al.^(22)^ showed the usability of small detectors composed of low‐Z materials for kV dosimetry by comparing the response of microionization chambers in weak filtered beams from 20–250 kVp relative to ^60^Co. Scandurra and Lawford^(23)^ used a linear diode array for dose profile measurements at a kilovoltage cone‐beam CT attached to a linear accelerator.

In this work, the usability of common dosimeters for megavoltage photon beams in diagnostic CT radiation fields is systematically evaluated. Additionally, the results complement the hitherto existing beam quality correction factors for several detectors in the energy range from 125 kVp down to 50 kVp with filtrations commonly used in modern CT scanners.

## II. MATERIALS AND METHODS

### A. X‐ray beams

An Oldelft Simulix MC X‐ray device (Oldelft Benelux, Veenendaal, The Netherlands) with a Hofmann Selector MD HV generator (Hoffmann GmbH, Hamburg, Germany) and Varian G297 X‐ray tube in a Varian DO10 housing (Varian Medical Systems, Palo Alto, CA) was used for irradiation of all detectors. The X‐ray tube is mounted on a gantry in a focus‐isocenter‐distance of 100 cm with a collimator. This enables full control of focus‐surface distance, field size, and shape. Inherent filtering of the whole unit is 3.3 mm Al. Additional filters were used in a tray located 30 cm from focus, mounted at the collimator. The high voltage generator allows accelerating potentials ranging from 40 kVp up to 125 kVp.

In order to evaluate the performance of various detectors in CT radiation fields, beam qualities similar to those common for CT devices were used. The total filtering was adjusted according to data provided by Siemens AG (Erlangen, Germany) with a mean value for the bowtie filter in a Siemens SOMATOM Sensation 64 CT unit. Table 1 lists the chosen beam qualities and the beam qualities listed in the CT scanner user guide, which are measured at minimal bowtie filter thickness.^(24)^ The half‐value layer (HVL) was measured with a PTW T60004 diode (PTW‐Freiburg, Germany) for kV dosimetry inserting additional Al absorbers in steps of 0.1 mm into the tray until the initial reading is halved.

**Figure 1:**
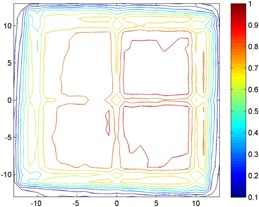
Relative dose distribution for the Oldelft Simulix MC, measured with the PTW 2D‐Array at 120 kVp and 800 mAs and a field size of $20\times 20\;{\text{cm}}^{2}$ and normalized to the maximum dose within the field. The physical crosshair and outer field size marking wires of the collimator box are clearly visible. The upper right quadrant of the radiation field, centered at x=5 cm and y=5 cm, has been identified as most homogeneous, in which subsequent measurements were carried out.

A CT radiation field is typically composed of a rotating collimated fan beam and may be moved along the craniocaudal axis for the actual scan length, resulting in a rectangular rotating radiation field. To eliminate differences in angular response of each detector, a stationary field was used. The X‐ray radiation field was collimated to a 20 cm×20 cm field in a focus distance of 100 cm. The manufacturer‐defined reference point of each detector was placed in this plane. Since the dose distribution of the radiation field is not completely uniform, a specific area of interest providing a flat homogeneous dose distribution was identified using the PTW 2D‐Array (Fig. 1), which is located at the upper right quadrant of the radiation field centering at x=5 cm and y=5 cm. The detectors were positioned with their reference points located at the center of this homogeneous field to minimize the influence of volume‐related weighted dose averaging.

**Table 1:** The X‐ray beam qualities used in this study. Each beam quality is listed with tube potential, measured half‐value‐layer (HVL) Al, and the corresponding air‐kerma beam quality correction factor PQ for the PTW 60004, used as reference for cross calibration. The beam classification code according to the IEC 61267 and the minimal HVL (${\text{HVL}}_{\text{Siemens}}$) provided by the manufacturer were added where appropriate

*IEC 61267 code*	*Peak Potential (kVp)*	*HVL(Al) (mm)*	*HVL_Siemens_(Al) (mm)*	*P_Q_*
RQA 3	50	3.8		1.020
RQA 5	70	6.8		1.000
	80	7.1	6.8	0.996
	100	8.5	8.1	0.979
	120	10.4	9.1	0.975
RQA 9	125	11.6		0.980

### B. Detectors

In this study, three Si‐diodes, a pencil‐type ionization chamber, five thimble ionization chambers, six small volume pinpoint chambers, and three parallel plate chambers (including a 2D‐Array) were investigated. The specifications of all investigated detectors are listed in 2, 3, 4 based on data from the manufacturers.^25^, ^26^ All investigated detectors, except the parallel plate chambers, possess rotation‐symmetric designs, which is crucial for CT dosimetry. The parallel plate chambers were included in this study for comparison with previous studies.^20^, ^21^

These detectors were chosen also by taking into consideration their general suitability according to DIN 6809‐4 and DIN 6809‐5, which state that the sensitive volume of parallel plate chambers or compact chambers should not exceed $1\;{\text{cm}}^{3}$ when used for X‐ray dosimetry in this energy range. For CTDI measurements, only pencil‐type chambers are recommended.^27^, ^28^ In general, plane parallel detectors are not suited for rotating radiation fields. Since certain measurement tasks, such as acquiring percent depth‐dose curves or the localization scan at the beginning of a CT examination, are done with a stationary X‐ray tube, these detectors can still be used. General physical properties of the 2D‐Array seven29 have been investigated and described by Poppe et al.^29^, ^30^, ^31^

The PTW 60004 diode was used with a PTW DIADOS electrometer which was included in the original calibration process.^(32)^ All remaining detectors were used in combination with a PTW UNIDOS electrometer in electrical readout mode, so no further detector specific calibration factors were applied.

**Table 2:** Thimble ionization chambers and the pencil type chamber used in this study, including the calculated maximum variation in energy response and a short summary of the physical properties and geometry

			*Energy Response Variation*	
*Name*	*Model Code*	*Specifications*	*In Free Air*	*2 cm Water*
0.125 Semiflex	PTW 31010	Semiflexible ionization chamber with 0.55 mm PMMA, 0.15 mm graphite wall (total wall area density $78\;\text{mg}\;{\text{cm}}^{-2}$) and 0.125 cm^3^ sensitive volume, 1.1 mm diameter Al central electrode	2.9%	10.4%
0.3 Semiflex	PTW 31013	Semiflexible ionization chamber with 0.55 mm PMMA, 0.15 mm graphite wall (total wall area density $78\;\text{mg}\;{\text{cm}}^{-2}$) and 0.3 cm^3^ sensitive volume, 0.9 mm diameter Al central electrode	4.1%	12.2%
Compact Chamber 08	IBA CC 08	Semiflexible ionization chamber with $70\;\text{mg}\;{\text{cm}}^{-2}$ air‐equivalent plastic C552 wall, 0.08 cm^3^ sensitive volume and C552 central electrode	9.4%	11.6%
Compact Chamber 13	IBA CC 13	Semiflexible ionization chamber with $70\;\text{mg}\;{\text{cm}}^{-2}$ air‐equivalent plastic C552 wall, 0.13 cm^3^ sensitive volume and C552 central electrode	3.2%	8.1%
Compact Chamber 13S	IBA CC 13‐S	Semiflexible ionization chamber with $154\;\text{mg}\;{\text{cm}}^{-2}$ air‐equivalent plastic C552/PEEK wall, 0.13 cm^3^ sensitive volume and C552 central electrode	3.4%	11.8%
CTDI chamber	PTW 30009	Pencil‐type ionization chamber with 1 mm PMMA wall (total wall area density $119\;\text{mg}\;{\text{cm}}^{-2}$) and 3.14 cm^3^ sensitive volume, 3 mm outer and 2 mm inner diameter Al tube central electrode	2.3%	–

**Table 3:** Parallel plate ionization chambers and diodes used in this study, including the calculated maximum variation in energy response and a short summary of the physical properties and geometry

			*Energy Response Variation*	
*Name*	*Model Code*	*Specifications*	*In Free Air*	*2 cm Water*
kV diode	PTW 60004	Si diode for 40 – 150 kVp X‐rays, calibrated in different radiation qualities for air kerma by manufacturer	2.5%	2.5%
Capsuled diode	PTW 60008	p‐type Si diode with $1\;{\text{mm}}^{2}\times 2.5\;\mu m$ sensitive volume and 2.2 mm water‐equivalent window thickness	35.0%	37.7%
Uncapsuled diode	PTW 60012	p‐type Si diode with $1\;{\text{mm}}^{2}\times 2.5\;\mu m$ sensitive volume and 0.7 mm water‐equivalent window thickness	23.7%	30.6%
Markus chamber	PTW 23343	Parallel plate ionization chamber with 0.03 mm PE entrance foil and 0.055 cm^3^ sensitive volume	19.0%	3.4%
Roos chamber	PTW 34001	Parallel plate ionization chamber with 1.0 mm PMMA entrance window and 0.35 cm^3^ sensitive volume	15.4%	2.5%
2D‐Array	PTW 10024	27×27 ionization chambers of 5 mm×5 mm×5 mm dimension and 0.125 cm^3^ sensitive volume	–	15.4%

**Table 4:** Small volume ionization chambers and diodes used in this study, including the calculated maximum variation in energy response and a short summary of the physical properties and geometry

			*Energy Response Variation*	
*Name*	*Model Code*	*Specifications*	*In Free Air*	*2 cm Water*
PinPoint 06	PTW 31006	Small volume ionization chamber with 0.57 mm PMMA, 0.09 mm graphite wall (total wall area density $85\;\text{mg}\;{\text{cm}}^{-2}$) and 0.015 cm^3^ sensitive volume, steel central electrode	18.9%	20.8%
PinPoint 14	PTW 31014	Small volume ionization chamber with 0.66 mm PMMA, 0.09 mm graphite wall (total wall area density $89\;\text{mg}\;{\text{cm}}^{-2}$) and 0.015 cm^3^ sensitive volume, 0.3 mm Al central electrode	8.6%	4.5%
PinPoint 15	PTW 31015	Small volume ionization chamber with 0.66 mm PMMA, 0.09 mm graphite wall (total wall area density $89\;\text{mg}\;{\text{cm}}^{-2}$) and 0.03 cm^3^ sensitive volume, 0.3 mm Al central electrode	8.3%	4.8%
PinPoint 16	PTW 31016	Small volume ionization chamber with 0.66 mm PMMA, 0.09 mm graphite wall (total wall area density $89\;\text{mg}\;{\text{cm}}^{-2}$) and 0.01 cm^3^ sensitive volume, 0.3 mm Al central electrode	12.4%	20.9%
Compact Chamber 01	PTW CC 01	Small volume ionization chamber with $88\;\text{mg}\;{\text{cm}}^{-2}$ air‐equivalent plastic C552 wall, 0.01 cm^3^ sensitive volume and steel central electrode	15.4%	18.4%
Compact Chamber 04	PTW CC 04	Small volume ionization chamber with $70\;\text{mg}\;{\text{cm}}^{-2}$ air‐equivalent plastic C552 wall, 0.13 cm^3^ sensitive volume and C552 central electrode	2.7%	11.9%

### C. Relative detector response

The relative detector response (RDR) of an ionization chamber or dosimetry diode was determined according to the previously published method.^20^, ^21^ The RDR for a detector of interest RDR(E)_x_ at a particular beam quality, E, was determined as follows:

(2) $$RDR{\left(E\right)}_{x}=P_{Q}{\left(E\right)}_{f}\cdot \frac{M{\left(E\right)}_{\text{Ref}}}{M{\left(E\right)}_{x}}$$

where $P_{Q}{\left(E\right)}_{Ref}$ is the air‐kerma beam quality correction factor (see Table 1) for the PTW 60004 reference diode provided by the manufacturer within the calibration sheet, $M{\left(E\right)}_{Ref}$ is the electrometer reading for the PTW 60004 reference diode, and $M{\left(E\right)}_{x}$ is the reading for the ionization chamber or diode under investigation. If the measured beam quality did not match the provided values, $P_{Q}{\left(E\right)}_{\text{Ref}}$ was calculated using the two closest given qualities and a short range linear interpolation. Since all measured data were normalized to the reading at 70 kVp and taken in a close timely manner to their normalization readout, additional corrections like pressure and temperature correction were not applied, but monitored. This peak potential was chosen because the PTW 60004 reference diode was calibrated at this beam quality, and a correction factor has to be applied for every other quality. The maximum variation in energy response for this diode is 2.5% (Table 3).

The RDR in air was measured with each detector listed in 2, 3, 4, except for the 2D‐Array. Measurements in free air for this detector could not be performed due to the inherent detector housing material (water‐equivalent window thickness of 5.9 mm). Similarly, the RDR was measured at 2 cm depth in a water tank for all waterproof ionization chambers. Since the PTW 60004 reference diode and the 2D‐Array are not waterproof, the measurements of these detectors were performed in a Solid Water phantom made from RW3. RW3, as well as PMMA (the standard phantom material in X‐ray beams up to 100 kVp according to DIN 6809‐4) are not recommended for measurements of depth dose data, but relative measurements at a fixed depth are still feasible.^27^, ^33^

The uncertainties associated with a comparable measurement setup as used in this study have been analyzed by Hill et al.^(20)^ Uncertainty components attributed to the used detector, phantom material, movement of phantom, dose rate variations and linearity in output of the X‐ray tube, temperature, and pressure stability were taken into account. The overall uncertainty amounts to 1.5%. In this study, a monitoring ionization chamber was used to control the output from the X‐ray unit. Less than 0.2% variation in dose rate and linearity of the tube was observed. Therefore, the main uncertainty with the RDR measurement can be attributed to the calibration process and the used correction factor, which is provided by the manufacturer, and stated at 5%.

In order to evaluate the influence of the different phantom materials, a comparison measurement was performed with the PTW 31010 and PTW 31013 Semiflex chambers. For these chambers, the RDR was measured in water as well as in the Solid Water phantom. The difference in RDR was smaller than 2.6% for the PTW 31010 Semiflex chamber and smaller than 2.3% for the PTW 31013 Semiflex chamber, which is within the uncertainty of this measurement.

## III. RESULTS & DISCUSSION

The RDR calculated according to Eq. (2) for the thimble ionization chambers and the pencil‐type chamber, normalized to the response at 70 kVp, are presented in 2, 3, showing the RDR in air and water respectively. The RDR for the parallel plate ionization chambers and the 2D‐Array are shown in 4, 5. Similarly, 6, 7 show the RDR in air and water for the ionization chambers with small sensitive volume. The variations in energy response over the entire investigated energy region for each detector are listed in 2, 3, 4.

**Figure 2:**
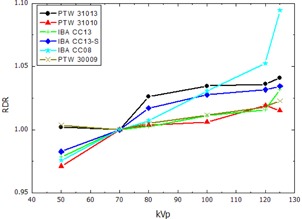
Relative detector response (RDR) of the investigated thimble ionization chambers and the pencil chamber in free air.

**Figure 3:**
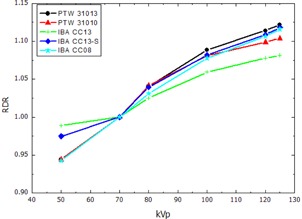
Relative detector response (RDR) of the investigated thimble ionization chambers and the pencil chamber at 2 cm depth in water.

**Figure 4:**
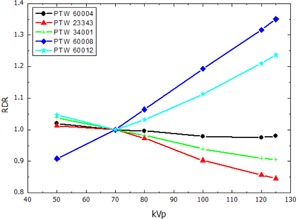
Relative detector response (RDR) of the investigated parallel plate ionization chambers and diodes in free air.

**Figure 5:**
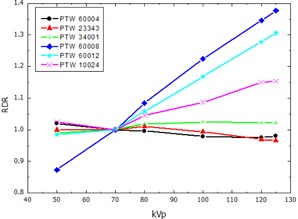
Relative detector response (RDR) of the investigated parallel plate ionization chambers, detector array, and diodes at 2 cm depth in water.

**Figure 6:**
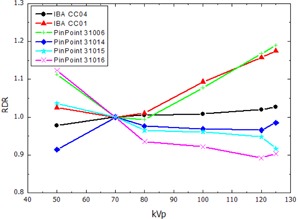
Relative detector response (RDR) of the investigated pinpoint ionization chambers in free air.

**Figure 7:**
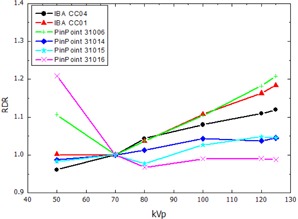
Relative detector response (RDR) of the investigated pinpoint ionization chambers at 2 cm depth in water.

### A. Thimble and pencil‐type ionization chambers

The RDR for the thimble ionization chambers were found to decrease with decreasing energy both in free air and in water (2, 3). This trend indicates the overresponse of the detector signal for lower energies compared to the PTW 60004 reference diode that can be attributed to the high‐Z components within the chambers, such as the inner electrodes, resulting in increased photoelectric effect interaction cross section relative to water or air. The amplification of this effect in water may be caused by the perturbation of the radiation field caused by the ionization chamber in terms of spectral and angular composition, as well as the conversion from air kerma in free air to air kerma in water.

The two Semiflex chambers, PTW 31010 and PTW 31013, and the Compact Chambers IBA CC08, CC13, and CC13‐S (IBA Dosimetry GmbH, Schwarzenbruck, Germany) show a similar RDR due to their very similar design. Major differences for these chambers are the length of the sensitive volume and the wall material. The PTW chamber wall is made of PMMA $\left(Z_{\text{mean}}=5.85,78\;{\text{mg cm}}^{-2}\right)$ and IBA uses C552 plastic $\left(Z_{\text{mean}}=7.11,70\;{\text{mg cm}}^{-2}\right)$. Therefore, the PTW chambers are equipped with a lower $Z_{\text{mean}}$, whereas the total wall density is higher than for IBA chambers. The ionization chambers with about 0.1 cm^3^ sensitive volume show the lowest variation in energy response. Especially the PTW 31010 Semiflex chamber (0.125 cm^3^) and IBA CC13, as well as IBA CC13‐S (0.13 cm^3^) Compact Chambers, all having nearly the same sensitive volume, showed very similar energy response variation in free air of 2.9%, 3.2%, 3.4%, respectively. This implies that the combination of lower Z and higher density in case of the PTW Semiflex chambers results in a similar photoelectric absorption for chambers of comparable volume. The results for the Semiflex chamber PTW 31013 are consistent with the results from the previous study by Hill et al.^(20)^ who reported a variation of 11% for an energy range up to 280 kVp.

Since the CTDI pencil‐type ionization chamber PTW 30009 is not waterproof and no suitable adapter for the Solid Water phantom was available, only measurement in free air was possible. A direct comparison to the manufacturer‐provided correction factors is not possible since the CTDI chamber was calibrated at a different energy. Nevertheless the variation in energy response of 2.3% found in this study is comparable to the manufacturer‐provided value of 3%.

The largest variation in RDR in this detector group was found for the Compact Chamber IBA CC08. With the smallest sensitive volume of 0.08 cm^3^ in this group this chamber shows the properties similar to the small volume chambers discussed later.

### B. Parallel plate ionization chambers and dosimetry diodes

In this study, an increasing RDR in free air of 19% variation for the PTW 23343 Markus chamber and 15.4% for the PTW 34001 Roos chamber (Fig. 4, Table 3) was found. A relatively flat RDR with 3.4% variation for the Markus chamber and 2.5% for the Roos chamber in solid water was found. Previous studies showed deviating results. Hill et al.^(20)^ reported a rapidly increasing RDR for the Markus chamber as the energy of the incident X‐ray beam decreases from 280 kVp down to 50 kVp. Contrary to this, Li et al.^(21)^ found a decreasing RDR in the range from 300 kVp down to 200 kVp, which was not confirmed by Hill and colleagues. However, from 200 kVp down to 50 kVp, the Li study reported an increasing RDR. Direct comparison of these results in the literature is difficult due to discrepancies in the experimental setup and the normalization of the calculated values in these three studies. One reason for the observed differences could be the different effective Z of the entrance window of the Markus type chambers. Hill et al. used a Markus chamber with Mylar (PET) foil, whereas, in our study, a Markus chamber with PE foil was investigated that is expected to show weaker energy dependence. Additionally, the used X‐ray field varied in size ($10\times 10\;{\text{cm}}^{2}$ in Li et al, 6 cm diameter in Hill et al., and $20\times 20\;{\text{cm}}^{2}$ in our study), as well as in SSD (50 cm in Li et al., 30 cm in Hill et al., and 100 cm in our study), and spectral composition (e.g., HVL(Al) of the 100 kVp beam: 2.43 mm in Li et al., 3.9 mm in Hill et al., and 8.5 mm in our study).

The Roos type chamber was only used by Hill and colleagues and showed a similar behavior like the Markus chamber which could be confirmed here. The Hill study showed already the accuracy of the percent depth‐dose curves measured with both the Markus and Roos type chambers.^(20)^ This indicates that the beam hardening effect causes only small changes in the energy response of the investigated detectors.

Both investigated dosimetry diodes PTW 60008 and PTW 60012 showed stronger energy dependence in free air as well as in water than the air cavity based detectors. This may be due to the optimization of the construction for high MeV beams, like the introduction of additional guarding electrodes.^(34)^ Since the rotational symmetry of both MV diodes is a desirable property, their energy dependence limits their use to well‐known radiation fields in which the energy spectrum is known or does not vary significantly.

### C. Small volume ionization chambers

Comparison between the small volume ionization chambers IBA CC01, CC04, PTW 31006, 31014, 31015, and 31016 with the larger thimble chambers shows in general a larger RDR variation for the smaller chambers. This is caused by the increasing ratio of high‐Z materials like Al or C in the electrodes compared to the small air cavity and, thus, more energy deposition through photoelectric effect. Additionally, the overall design of the chamber has a large impact on the results due to the limited size of the chamber, and may lead to an opposite effect.

The largest energy dependency in this group of detectors was found for the pinpoint chambers PTW 31006 (18.9% variation in free air, 20.8% in water) and IBA CC01 (15.4% in free air, 18.4% in water). This is attributed to the steel central electrode in these chambers, perturbing the radiation field due to the increased photoelectric cross section. The pinpoint chambers PTW 31014, PTW 31016 and especially the PTW 31015 and IBA CC04, which have larger sensitive volumes and are equipped with Al central electrodes, do not contain as much high‐Z material. Therefore, a lower overresponse for low‐energy beams compared to the steel electrode pinpoint chambers IBA CC01 and PTW 310006 is expected and was found in free air (Fig. 6), as well as in water (Fig. 7).

The PTW 31016 showed the greatest variation of relative detector response. Based on the manufacturer's data, this chamber is optimized for a 3D response in small MV beams. In the intended application fields described in this work, this seems not to be advantageous.^(25)^ These effects regarding the photon energy spectra, especially for different water depths, are subject of further investigation.

### D. 2D‐Array

The PTW 2D‐Array seven29 showed a larger RDR variation than the single ionization chambers with comparable volume (PTW 31010, IBA CC13, IBA CC13S). This may be due to the copper‐based electrode matrix, which is used as electrode in this detector. However, the RDR of the 2D‐Array with 15% variation is comparable to 8%–12% variations for the single chambers in the whole investigated energy region. The disadvantage of the PTW 2D‐Array is its nonrotational symmetric design of the single chambers. Nevertheless, the 2D‐Array can be very useful in nonrotating radiation fields, such as the overview scan before a CT examination or for measurements in service mode with a fixed X‐ray tube. It enables the acquisition of the whole two‐dimensional beam profiles in a single measurement, similar to radiographic films.

## IV. CONCLUSIONS

It has been demonstrated that the thimble ionization chambers PTW 31010 and PTW 31013, as well as the IBA Compact Chambers of comparable sensitive volume IBA CC13 and IBA CC13S, with a maximum variation in RDR in free air of 5% are well suited for dosimetry in X‐ray fields of computed tomography and CBCT units, such as those integrated to linear accelerators for image‐guided radiation therapy. If higher spatial resolution is needed, Compact Chamber IBA CC04 or PTW 31015 or PTW 31016 PinPoint chambers with Al central electrodes can also be used. Energy response of these detectors has been analyzed by the method proposed by Li et al.^(21)^ and Hill et al.^(20)^ Besides the general characterization of the detectors spectral sensitivity, the RDR can be used as a correction factor for kV dosimetry in radiation fields of similar HVL. The rotational symmetry of these detectors is of major advantage for CT dosimetry and, in conclusion, absorbed dose profile measurements as well as point dose measurements in a suitable phantom for estimation of organ doses are enabled.

Dosimetry diodes PTW 60008 and PTW 60012 are of limited use in this low‐energy range due to their optimization in designs for high MV beams.

Parallel plate chambers PTW 23343 and PTW 34001 showed a similar RDR to previous studies. In addition to these chambers, the PTW 10024 2D‐Array was investigated and a comparable variation in detector response in this energy range was found. Therefore, it is worthwhile to investigate the application potential of such 2D‐Arrays for nonrotating irradiations.

## ACKNOWLEDGMENTS

This study was funded by the Bundesministerium fuer Bildung und Forschung through the grant 03NUK008A. See www.helmholtz‐muenchen.de/kvsf/ for further information.

## Supporting information

Supplementary Material

## References

[1] Schlattl H , Zankl M , Becker J , Hoeschen C . Dose conversion coefficients for CT examinations of adults with automatic tube current modulation. Phys Med Biol. 2010;55(20):6243–61. 20885020 10.1088/0031-9155/55/20/013

[2] Schlattl H , Zankl M , Petoussi‐Henss N . Organ dose conversion coefficients for voxel models of the reference male and female from idealized photon exposures. Phys Med Biol. 2007;52(8):2123–45. 17404459 10.1088/0031-9155/52/8/006

[3] Schlattl H , Zankl M , Hausleiter J , Hoeschen C . Local organ dose conversion coefficients for angiographic examinations of coronary arteries. Phys Med Biol. 2007;52(15):4393–408. 17634640 10.1088/0031-9155/52/15/003

[4] Williams G , Zankl M , Abmayr W , Veit R , Drexler G . The calculations of dose from external photon exposures using reference and realistic human phantoms and Monte Carlo methods. Phys Med Biol. 1986;31(4):449–52. 3737684 10.1088/0031-9155/31/4/010

[5] Medical electrical equipment, part 2‐44: Particular requirements for the basic safety and essential performance of X‐ray equipment for computed tomography. International Standard IEC 60601‐2‐44, 3rd ed Geneva: IEC; 2009.

[6] European Guidelines on quality criteria for computed tomography. Report EUR 16262. Brussels: European Commission; 1999.

[7] U.S. Food and Drug Administration. Computed tomography (CT) equipment. Code of Federal Regulations, Silver Spring, MD: FDA; 2008.

[8] U.S. Food and Drug Administration. Diagnostic X‐ray systems and their major components; amendments to performance standard. Final rule. Federal Register. 1984; 49(171):34698–714. 10267667

[9] Deutsches Institut fuer Normung E.V. (DIN). Clinical dosimetry – Part 3: Diagnostic radiology [in German]. DIN 6809‐3. Berlin: Beuth Verlag; 1990.

[10] Geleijns J , Salvado Artells M , de Bruin PW , Matter R , Muramatsu Y , McNitt‐Gray MF . Computed tomography dose assessment for a 160 mm wide, 320 detector row, cone beam CT scanner. Phys Med Biol. 2009;54(10):3141–59. 19420423 10.1088/0031-9155/54/10/012 PMC2948862

[11] Mori S , Endo M , Nishizawa K , Murase K , Fujiwara H , Tanada S . Comparison of patient doses in 256‐slice CT and 16‐slice CT scanners. Br J Radiol. 2006;79(937):56–61. 16421406 10.1259/bjr/39775216

[12] Dixon RL . A new look at CT dose measurement: beyond CTDI. Med Phys. 2003;30(6):1272–80. 12852553 10.1118/1.1576952

[13] Dixon RL and Boone JM . Dose equations for tube current modulation in CT scanning and the interpretation of the associated CTDIvol. Med Phys. 2013;40(11):111920. 24320453 10.1118/1.4824918

[14] Liebmann M , Luellau T , Feltes M , Poppe B , von Boetticher H . Comparison of different methods for measuring CT dose profiles with a new dosimetry phantom. In: 13th International Radiation Protection Association Congress, Glasgow, Scotland, 2012.

[15] Liebmann M , Luellau T , Uhlig CH , et al. [Development of a multi‐purpose phantom for CT dosimetry] [in German]. In: Abstracts / Medizinische Physik 2011. Vienna; 2011.

[16] Martin CJ , Gentle DJ , Sookpeng S , Loveland J . Application of gafchromic film in the study of dosimetry methods in CT phantoms. J Radiol Prot. 2011;31(4):389–409. 22089894 10.1088/0952-4746/31/4/001

[17] Dixon RL and Ballard AC . Experimental validation of a versatile system of CT dosimetry using a conventional ion chamber: beyond CTDI100. Med Phys. 2007;34(8):3399–413. 17879802 10.1118/1.2757084

[18] Mori S , Endo M , Nishizawa K , et al. Enlarged longitudinal dose profiles in cone‐beam CT and the need for modified dosimetry. Med Phys. 2005;32(4):1061–69. 15895591 10.1118/1.1877852

[19] Hill R , Healy B , Holloway L , Kuncic Z , Thwaites D , Baldock C . Advances in kilovoltage x‐ray beam dosimetry. Phys Med Biol. 2014;59:R183–R231. 24584183 10.1088/0031-9155/59/6/R183

[20] Hill R , Mo Z , Haque M , Baldock C . An evaluation of ionization chambers for the relative dosimetry of kilovoltage X‐ray beams. Med Phys. 2009;36(9):3971–81. 19810470 10.1118/1.3183820

[21] Li XA , Ma CM , Salhani D . Measurement of percentage depth dose and lateral beam profile for kilovoltage X‐ray therapy beams. Phys Med Biol. 1997;42(12):2561–68. 9434308 10.1088/0031-9155/42/12/019

[22] Snow JR , Micha JA , DeWerd LA . Microionization chamber air‐kerma calibration coefficients as a function of photon energy for X‐ray spectra in the range of 20‐250 kVp relative to 60Co. Med Phys. 2013;40(4):041711. 23556881 10.1118/1.4794491

[23] Scandurra D and Lawford E . A dosimetry technique for measuring kilovoltage cone‐beam CT dose on a linear accelerator using radiotherapy equipment. J Appl Clin Med Phys. 2014;15(4):80–92. 10.1120/jacmp.v15i4.4658 PMC5875512 25207398

[24] Siemens AG . Siemens SOMATOM Sensation 64 Users Guide. Malvern, PA: Siemens AG.

[25] Physikalisch‐Technische Werkstaetten Dr . Pychlau GmbH (PTW). Ionizing radiation detectors. Freiburg, Germany: PTW; 2011.

[26] IBA Dosimetry GmbH. Detectors for relative and absolute dosimetry: ionization chambers and diode detectors. Schwarzenbruck, Germany: IBA GmbH; 2009.

[27] Deutsches Institut fuer Normung E.V. (DIN). Clinical dosimetry – Application of X‐rays with peak voltages between 10 and 100 kV in radiotherapy and soft tissue diagnostics [in German]. DIN 6809‐4. Berlin: Beuth Verlag; 1988.

[28] Deutsches Institut fuer Normung E.V. (DIN). Clinical dosimetry – Application of X‐rays with accelerating potentials from 100 to 400 kV in radiation therapy [in German]. DIN 6809‐5. Berlin: Beuth Verlag; 1996.

[29] Poppe B , Blechschmidt A , Djouguela A , et al. Two‐dimensional ionisation chamber arrays for IMRT plan verification. Med Phys. 2006;33(4):1005–15. 16696477 10.1118/1.2179167

[30] Poppe B , Rubach A , Harder D , Willborn K . A method to increase the resolution of IMRT plan verification with a two‐dimensional ionisation chamber array [abstract]. Med Phys. 2006;33(4):2054–55. 10.1118/1.2179167 16696477

[31] Poppe B , Atung Z , Chofor G , et al. Resolution and sensitivity of two‐dimensional ionization chamber‐arrays (PTW type 10024) [in German]. Z Med Phys. 2005;15(4):287–91. 16422358 10.1078/0939-3889-00261

[32] Physikalisch‐Technische Werkstaetten Dr. Pychlau GmbH (PTW). DIADOS E diagnostic dosemeter. Freiburg, Germany: PTW GmbH; 2001.

[33] Hill R , Kuncic Z , Baldock C . The water equivalence of solid phantoms for low energy photon beams. Med Phys. 2010;37(8):4355–63. 20879595 10.1118/1.3462558

[34] Djouguela A , Griessbach I , Harder D , et al. Dosimetric characteristics of an unshielded p‐type Si diode: linearity, photon energy dependence and spatial resolution. Z Med Phys. 2008;18(4):301–06. 19205300 10.1016/j.zemedi.2008.06.007

