# Supplementary figures and images for: Computed tomography dosimetry with high‐resolution detectors commonly used in radiotherapy — an energy dependence study
Source: J Appl Clin Med Phys. 2015 Sep 8;16(5):396–407. doi: 10.1120/jacmp.v16i5.5302 (PMC5690150; doi:10.1120/jacmp.v16i5.5302)

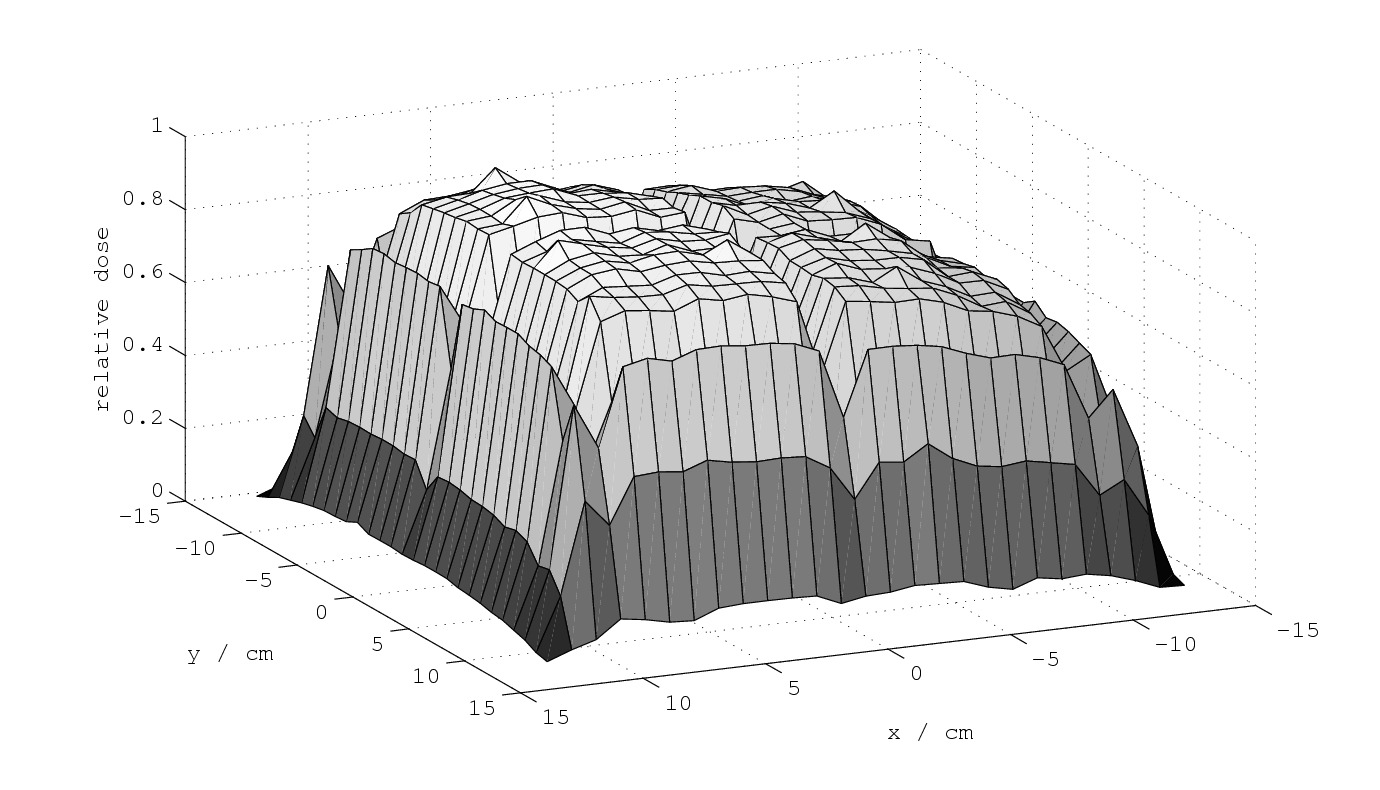

Supplement: Supplementary file 1 — Supplementary Material [file ACM2-16-396-s001.jpg]
